# Editorial: Women at the forefront of musculoskeletal aging science

**DOI:** 10.3389/fragi.2026.1855184

**Published:** 2026-04-30

**Authors:** Caglar Cosarderelioglu, Stefania Toselli, Ligiana Pires Corona

**Affiliations:** 1 Johns Hopkins University School of Medicine, Division of Geriatric Medicine and Gerontology, Baltimore, MD, United States; 2 Department for Life Quality Studies, University of Bologna, Bologna, Italy; 3 School of Applied Sciences, State University of Campinas, Limeira, Brazil

**Keywords:** handgrip strength, multimorbidity, muscle function, musculoskeletal aging, osteoarthritis, physical activity, sarcopenia, women in science

Musculoskeletal aging represents a key dimension of the aging process, with major consequences for physical function, mobility, and independence in later life. Its clinical manifestations are diverse, ranging from sarcopenia and osteoarthritis to broader impairments in physical performance. At the same time, the scientific community studying these conditions continues to reflect persistent gender disparities, with women still underrepresented in research worldwide.

This Research Topic was developed within this dual context: to advance understanding of musculoskeletal aging while also highlighting the contributions of women scientists in the field. The Research Topic brings together a mini review, a brief research report, and three original research articles, reflecting the breadth of current work in this area, from molecular mechanisms and diagnostic innovation to clinical epidemiology and behavioral science, while also drawing attention to the role of women both as researchers and as participants in musculoskeletal aging research. Importantly, several contributions within this Research Topic also include predominantly female cohorts or address sex-specific aspects of musculoskeletal aging, further reinforcing the relevance of a gender-informed perspective in this field.

The review by Damanti et al. *,* sets the stage for this Research Topic by offering a comprehensive overview of skeletal muscle aging. It begins by underscoring the importance of the musculoskeletal system and its underlying architecture, then moves through the structural and functional changes that occur with age, and finally examines the biological pathways that drive these processes and interact with one another. Importantly, the authors emphasize that muscle aging is not a single process, but rather the result of converging mechanisms involving inflammation, mitochondrial dysfunction, hormonal decline, and impaired satellite cell activity. These insights are particularly relevant for understanding sex-specific trajectories of muscle aging, especially in light of hormonal changes across the female lifespan.

Building on this mechanistic framework, Pus et al., investigate tensiomyography (TMG) as a novel tool for characterizing muscle contractile properties in older adults and for refining the identification of sarcopenia. TMG is a non-invasive mechanomyographic method that measures muscle mechanical responses directly over the muscle belly, offering insight into aspects of neuromuscular function. The authors show that sarcopenic individuals display distinct alterations in TMG-derived contractile parameters, including prolonged delay and contraction times and reduced radial displacement, with muscle- and sex-specific patterns. Clinically, this study draws attention to a key gap in current diagnostic approaches, which rely largely on indirect or late-stage markers of muscle impairment. TMG may therefore represent a useful complementary method for detecting subtler changes in muscle quality and contractile behavior in aging populations. In this context, the identification of sex-specific patterns further highlights the importance of gender-sensitive approaches in sarcopenia assessment. Similarly, addressing the challenge of assessment, Benatti de Oliveira et al., evaluated the reliability of the Gripwise digital dynamometer for measuring handgrip strength, a cornerstone metric in geriatric practice, in older Brazilian adults, comparing its performance with the widely used Saehan device. Although the authors found reliability for both instruments, they also observed significant differences in handgrip strength values between the two devices. Importantly, these differences affected the classification of dynapenia, with the Gripwise identifying more cases of low muscle strength than the Saehan. This study therefore raises a critical issue: device-dependent variability may influence the cut-off values used to define dynapenia and, in turn, affect clinical decision-making. In an era when portable technologies are increasingly incorporated into both research and routine care, these findings underscore the need for standardization and for device-specific, context-sensitive cut-offs. Such considerations are particularly relevant when assessing heterogeneous populations, including women for whom normative values and thresholds may differ. Beyond skeletal muscle, the interplay between musculoskeletal disease and systemic comorbidity is addressed by Bindoli et al. in their monocentric study, which examined 87 patients in a predominantly female cohort. Their work broadens our understanding of erosive hand osteoarthritis (EHOA), a severe condition that primarily affects postmenopausal women, directly impacting joint function and hand strength, by linking local joint damage to broader systemic factors. In this cohort, higher body mass index and osteoporosis were associated with more severe radiographic abnormalities, while FRAX-estimated fracture risk also correlated with radiographic severity. These findings support the view that EHOA may be better understood not simply as a localized degenerative disorder of the hand, but as a systemic and metabolically influenced condition. More broadly, this study reinforces a core principle of geriatric medicine: musculoskeletal disorders rarely occur in isolation, and their clinical burden is often amplified by multimorbidity. The predominance of women in this cohort further emphasizes the need to better define sex-specific risk profiles and disease trajectories in EHOA.

Finally, Hadfield et al., offer a complementary perspective by exploring the lived experiences of a small group of premenopausal women participating in a group-based exercise intervention. Their findings highlight the importance of enjoyment, social connection, and prior experiences in shaping physical activity behaviors. Notably, participants described positive, socially supportive exercise environments as key facilitators of sustained behavioral change, an insight that is highly relevant for the design of interventions across the life course, including in older populations. The authors emphasize the value of reshaping negative perceptions of exercise and fostering a sense of capability rather than performance to support long-term adherence. These findings also underline the value of integrating lived experiences into intervention design, particularly when aiming to improve health and preventing physical decline before menopause occurs.

Taken together, these contributions illustrate the complexity of musculoskeletal aging and the need to approach it from multiple angles. They also highlight several emerging themes. First, there is increasing recognition of the molecular processes that underlie age-related decline. Second, accurate and context-sensitive measurement remains a central challenge, particularly as new technologies are introduced into clinical practice. Third, musculoskeletal health must be understood within a broader biopsychosocial framework that includes comorbidities, lifestyle, and lived experience. Equally important is the perspective that frames this Research Topic. Visibility of women’s contributions is essential to fostering a more inclusive research environment and to inspiring future generations of scientists. Looking ahead, interdisciplinary and gender-aware research will be essential to deepen our understanding of musculoskeletal aging and to translate these insights into more effective and inclusive clinical strategies. In conclusion, this Research Topic advances our understanding of musculoskeletal aging across biological, clinical, and behavioral domains while underscoring the importance of inclusivity in scientific research, illustrating that women are at the forefront of muscle science, driving essential discoveries for healthy aging. As Topic Editors of *Women at the Forefront of Musculoskeletal Aging Science* in *Frontiers in Aging*, we hope this Research Topic will stimulate further research and deepen current understanding of musculoskeletal aging. We extend our sincere appreciation to all authors and reviewers for their valuable contributions to this Research Topic.

